# Regulatory and policy tools to address unproven stem cell interventions in Canada: the need for action

**DOI:** 10.1186/s12910-019-0388-4

**Published:** 2019-08-06

**Authors:** Timothy Caulfield, Blake Murdoch

**Affiliations:** Health Law Institute, Faculty of Law, University of Alberta, 468 Law Centre, Edmonton, Alberta T6G 2H5 Canada

**Keywords:** Stem cell therapies, Unproven therapies, Policy, Law, Regulation

## Abstract

**Background:**

The marketing of unproven direct-to-consumer stem cell interventions is becoming widespread in Canada. There is little evidence supporting their use and they have been associated with a range of harms. Canada has been slower to act against clinics offering these interventions than other jurisdictions, including the United States. Here, we outline the regulatory and policy tools available in Canada to address this growing problem.

**Main body:**

Health Canada’s regulations governing cell therapies are complex, but recent statements make it clear that Health Canada believes it has jurisdiction over many of the currently marketed stem cell interventions. Still, further regulatory clarity is needed from Health Canada, as are increased directed enforcement efforts on interventions that fall within their scope. The Competition Bureau, via the *Competition Act*, prohibits advertisers from making materially false or misleading promotional representations. The Competition Bureau could collaborate with the scientific community to analyze the claims of existing clinics in Canada, and impose sanctions upon those who breach the established standard. Professional regulators, including provincial colleges of physicians and surgeons, have considerable power over what products and services their members can offer. Every college of physicians in Canada requires, via policy and codes of ethics, that doctors maintain evidence-based practices. This requirement is incompatible with offering many unproven stem cell interventions. Litigation may be another tool, including the use of fraud, misrepresentation and/or negligence claims for failing to meet the required standard of care. Finally, political pressure on federal and provincial lawmakers could encourage changes to marketing, cell therapy and professional regulations that would allow a more comprehensive response.

**Conclusions:**

In sum, there are many existing tools that can be used to protect the public from unproven stem cell interventions. Increased bureaucratic will and grassroots efforts are needed in order to effect a positive policy response.

## Background

A range of stem cell interventions is now widely offered directly to consumers in the United States and Canada [1, 2]. These interventions have not been approved by the Food and Drug Administration [FDA] [3] or by Health Canada [4, 5]. In general, there is little good clinical evidence to support their use [6–8]. These procedures have also been associated with a range of harms [9]. A 2018 review of adverse effects resulting from unproven stem cell interventions [USCIs] noted 35 cases of “acute or chronic complications” or death, and this was solely what could be discerned from reports in the media and scientific literature [10]. As summarized by George Daley, Dean of Harvard Medical School, except for a few widely accepted procedures, such as the use of bone marrow transplantations to treat some cancers, “stem-cell–based interventions remain unproven and experimental” [11]. Despite this reality, “anecdotal reports supercede hard evidence, and an industry peddling stem-cell–based products has formed” [11].

The spread of clinics marketing these interventions can also lead to the financial exploitation of patients – including the problematic use of crowdfunding [12] – and may, over the long term, damage public trust in legitimate regenerative technologies, thus adversely impacting their future development and translation [13]. This is particularly so given that both the clinics offering these interventions and the associated media coverage often misleadingly portray these interventions as effective, routine and safe [6, 14–16] – thus creating false expectations regarding the state of the science and readiness for clinical use.

The proliferation of clinics offering USCIs has prompted calls for policy change and enforcement efforts [6, 15, 17–19]. In the US, the FDA has taken explicit steps, including warning clinics via announcements about increased enforcement of regulations and oversight of stem cell clinics [20], and subsequently seeking permanent injunctions against specific clinics [21]. As part of their enforcement efforts, FDA officials have also made strong public statements, noting that the USCI industry is “leveraging the field’s hype to push unapproved, unproven, illegal, and potentially unsafe products” and that “[t] his is putting patients’ health at risk” [22]. The Federal Trade Commission has fined clinics for using misleading advertising [23, 24] and published a document, entitled “Stemming Unproven Stem Cell Therapy Claims,” that provides recommendations for patients and providers [25]. Australia has taken similar measures [26–28]. In addition, medical licensing boards are becoming aware of this growing problem. The Medical Board of California, for example, is now investigating how best to regulate members offering these interventions [29]. However, in Canada the response from regulators has been relatively muted.

Here, we outline the various legal and regulatory tools that can be used to combat the spread of direct-to-consumer USCIs in Canada, and provide ways for both medical professionals and members of the general public to engage with this issue. There are many such tools, indicating that addressing the issue may, at least initially, be more a matter of bureaucratic and political will than one of reform.

## Main text

### Health Canada

Health Canada regulates various cell interventions under the Food and Drugs Act [30], and more specifically the Food and Drug Regulations [FD Regulations] [31], the Medical Device Regulations [MD Regulations] [32] or the Safety of Human Cells, Tissues and Organs for Transplantation Regulations [CTO Regulations] [33, 34]*.* While Health Canada’s jurisdiction to regulate will vary based on the nature of the relevant intervention, for many interventions in the growing stem cell market Health Canada has the jurisdiction to take some action.

Recently, Health Canada has made it clear that, except for established lymphohematopoietic transplants for certain forms of cancer, all autologous cell therapies are considered “drugs” under the *Food and Drugs Act* [4, 30, 35] and therefore require federal approval. Such approval has not been provided for the stem cell interventions offered at private clinics in Canada. Furthermore, in a May 2019 statement, Health Canada warned Canadians about the potential health risks associated with unauthorized and unproven stem cell therapies [35].

Given scope of this problem, these kinds of definitive statements about Health Canada’s role are clearly welcome. In reality, however, the relevant regulatory framework is complex and somewhat unclear [34, 36]. Interventions using significantly manipulated cells are regulated under the FD Regulations, the MD Regulations or both [31, 32]. Allogeneic interventions for homologous use involving cells that meet the definition of minimally manipulated – cells for which “the processing does not alter the biological characteristics that are relevant to their claimed utility” – are regulated under the CTO Regulations [33]**.** However, most direct-to-consumer USCIs are autologous rather than allogeneic – that is, they use the patient’s own cells. Interventions that use minimally manipulated autologous cells were previously believed by some to fall into a regulatory gap [34, 37]. These interventions had been considered by some to be part of the practice of medicine and, therefore, exempt from Health Canada’s more comprehensive regulatory framework (e.g., requiring clinical trials to support regulatory approval). However, Health Canada’s May 2019 policy position statement states that minimally manipulated autologous cell interventions are regulated as drugs under the FD Regulations [35].

The regulatory justification by which these interventions come to be defined as “drugs” and subject to the FD regulations is not explicitly stated, but this may be uncontroversial since the definition of drug within the *Food and Drug Act* encompasses “any substance or mixture of substances manufactured, sold or represented for use in … the diagnosis, treatment, mitigation or prevention of a disease, disorder or abnormal physical state, or its symptoms” [30].

Further regulatory clarity would help Health Canada solidify its position, and increased enforcement efforts are needed. The United States has crafted regulation applicable to autologous interventions using minimally manipulated cells, though it has not fully prohibited their use [34, 37, 38]. Relatively minor changes in Canada could be fruitful. Experts had previously suggested modifying the CTO Regulations to make them applicable to both allogeneic and autologous uses and to justify Health Canada’s claim to control over USCIs [37]. Under Health Canada’s new standard, clarifications in the FD Regulations as well as public announcements providing layman descriptions of the types of the interventions prohibited would be helpful. Moreover, Health Canada’s Compliance and Enforcement Division should send cease and desist letters to clinics offering USCIs over which it has clear jurisdiction, followed up by or in concert with initiating proceedings to impose sanctions. Such a move would stand as an important policy statement regarding the issues associated with the provision of unproven interventions, may motivate policy action by other regulatory actors and would serve as an opportunity to further inform the public regarding the safety and efficacy issues.

### The Competition Bureau

The Competition Bureau is the federal body responsible for enforcing the *Competition Act,* and it regulates the content of Canadian advertising. The *Competition Act* states that individuals and companies cannot knowingly or recklessly make promotional representations that are “false or misleading in a material respect” [39]. A representation is materially false or misleading when the “literal meaning or general impression conveyed could influence the ordinary consumer to buy or use the advertised product or service” [40].

In addition, performance claims must be supported by an “adequate and proper test” [40], which does not require methodological rigour equivalent to that of scholarly research [41]. This standard, which was created by judicial precedent, only requires objectively establishing that the product or service has an effect beyond chance [41]**.** This standard can narrow the scope of application of this regulatory tool, particularly since peer-reviewed scientific evidence is not required to support a marketing claim. Still, given the preliminary state of the science in the context of many of the currently marketed stem cell products, the Competition Bureau remains a valuable enforcement tool as it can be used to weed out statements that do not accord with existing evidence.

The Competition Bureau could start by analyzing the advertising claims of the 43 clinics identified in Turner’s 2018 study of Canadian clinics offering USCIs [2]**.** Following analysis, the Bureau could collaborate with the Canadian research community (e.g., via the Stem Cell Network [42]) and Health Canada to determine which claims are materially false or misleading. The Bureau could advise clinics to cease using false or misleading claims, and could impose sanctions under the *Competition Act* [39]*.* While the Competition Bureau can only prohibit clinics from using misleading advertising – and not the provision of unproven interventions – this would help to stop the spread of misinformation about USCIs, which may curtail public interest.

### Professional regulation

Provincial colleges of physicians and surgeons have considerable control over what products and services their members can offer. These powers generally also apply to regulators of other healthcare providers that have been shown to offer stem cell interventions, such as naturopaths [43]. Though our comments are also largely applicable to other health professions, we will focus on physicians, as they appear to be the most common purveyors of USCIs [2, 43].

Policies and standards requiring that doctors maintain evidence-based practices are present among every provincial college [44–47]. Provincial disciplinary tribunal decisions have reinforced the rule that medical doctors must have an evidence-based practice [48, 49]. Moreover, many colleges have highly restrictive rules about how members can advertise services, especially those that may fall outside of typical standard of care [50, 51]. There are also binding codes of ethics, such as the Canadian Medical Association’s Code of Ethics and Professionalism [CMA Code], which require members to put the best interests of their patients first, and to ensure fully informed consent for treatment [52]. Recent additions to the CMA Code also clearly require members to “[r] ecommend evidence-informed treatment options” and to maintain professional integrity in a manner “consistent with evidence-informed decision-making” [52]**.** Doctors offering USCIs and advertising them as effective are likely in breach of these standards. While legislation exist in Ontario, Manitoba, Alberta and British Columbia directing colleges not to discipline members merely for employing “non-traditional” interventions unless the risk is greater than standard of care [53–56], these provisions do not override the requirements to have an evidence-based practice and to obtain full informed consent. Interpreting this “non-traditional” intervention exemption as removing the need for an evidence-based approach would effectively allow the colleges to abdicate their responsibilities as a self-regulating health profession. Of course, informed consent in the context of many USCIs would require disclosing the equivocal nature of the evidence using language that would often conflict with the claims made on clinic websites.

Despite the existence of policies requiring evidence-based practice, there is no indication that Canadian medical colleges, or colleges of other health practitioners for that matter, have taken significant action against USCIs. The College of Physicians and Surgeons of Alberta, for example, has even created draft regulations to set safety standards that would support and legitimize USCIs [57]. Colleges have been granted the power and responsibility to oversee the administration of evidence-based medicine to the public specifically because their membership, rather than legislators, have the greatest relevant expertise. Colleges should engage in the enforcement of their own rules and standards, notifying all members offering USCIs in a manner that conflicts with professional norms and standards to cease and desist. Justifications relating to Health Canada’s clear position that these therapies are unapproved would be useful to bolster such notices. In addition, there may be times when health professionals could be ethically required to report unprofessional USCI activity by fellow members to their respective self-regulators, in keeping with the CMA Code [52].

As mentioned, given that medical services such as minor surgery are typically considered part of medical practice, Health Canada’s position could be seen as government overreach. However, self-regulation is not a professional right, but rather a legal privilege granted by the state to experts in a field who should be the most knowledgeable and prudent individuals to set and enforce highly specialized policy. When there is clear evidence that self-regulating bodies are not taking action – for whatever reason – to protect the public interest, this triggers a need to consider other forms of regulatory oversight.

### Litigation

Litigation is another legal avenue which could prove useful to individuals who have been misled or experienced harm from USCIs or their advertising. False advertising claims under the *Competition Act,* and via common law fraud and misrepresentation, could disincentivize clinics from advertising or offering USCIs [39]. The standards for such claims would be similar to those of the Competition Bureau.

There is also the possibility of litigation on the basis of negligence for failing to meet the standard of care required of a health professional. Canadian physicians have an obligation to practice to the standard of a “prudent and diligent doctor in the same circumstances” [58]. This standard is arguably incompatible with non-evidence based interventions [59]. That said, the legal doctrine of the respected minority can allow healthcare practitioners to “depart from generally approved professional guidelines … by adopting newer, but non-mainstream practices” while not necessarily being negligent in cases where harm results [59, 60]. Yet, this defence relies on the practitioner having obtain full informed consent from the patient, an act that for most USCIs would include admitting there is no evidence the treatment works, that it is unapproved by Health Canada and that, often, there are better alternatives [59]. A clinic’s marketing materials alone could be so misleading as to the efficacy of USCIs that they make informed consent extremely challenging, as it would require clinicians to contradict their publicly-stated claims [16]. Successful litigation on the basis of negligence is potentially viable in cases where marketing claims made to the plaintiff are scientifically indefensible.

Notably, most litigation, especially when successful, could have the further positive effect of garnering public attention for these problematic activities, potentially spurring policy change. Litigation can also create precedent for future legal actions. That being said, the possibility of settlements that include nondisclosure agreements binding litigants to confidentiality could negatively impact the public’s access to information about the harms and ineffectiveness of USCIs.

### Advocacy

As noted, stopping the spread of USCIs is, in part, a matter of will on the part of the relevant regulatory bodies. Pressuring the legislative branch of government could help to spur on the institutions we mention above. For example, it would be possible for legislators to broaden Health Canada’s purview and bolster the legal justification for its claim to control over the approval of all stem cell interventions involving cells which are minimally manipulated. Moreover, provincial governments could also compel professional regulators to act to prohibit their membership from offering direct-to-consumer USCIs.

One area of broad relevance to public health in which lobbying could be effective is in relation to marketing claims, and specifically the noted judicial precedent allowing performance claims supported only by unscientific evidence [41]. Legislative change requiring methodologically robust and/or peer reviewed scientific evidence to support performance claims, along with enforcement of such a policy, would benefit not only those with the potential to be duped by USCIs but also those vulnerable to a wide variety of other unproven products and interventions.

In order to advocate for changes, influential commentators, institutional actors, and members of the general public need to engage with legislators. Advocating directly to provincial and federal lawmakers could be an effective strategy. Contacting federal and provincial health ministers, as well as local members of parliament and of provincial legislatures, with complaints, concerns and suggestions would be a good starting point. Individuals who have experienced misleading marketing and/or harmful treatments may have the most powerful and effective narratives around which to coalesce. However, the scientific community and institutional actors must also engage. The International Society for Stem Cell Research took an important step in 2018 by sending a detailed appeal to the federal Minister of Health, requesting several changes to Health Canada’s Cell Therapy Products guidance to “rein in unscrupulous clinics marketing unproven therapies as stem cell treatments” [61]. This has arguably already contributed to Health Canada’s more robust recent policy position statement [35], and is a solid foundation upon which to build with further advocacy.

There will be resistance to these advocacy efforts. Purveyors of USCIs who stand to profit from their use would likely resist changes, and those patients who experience the placebo effect or otherwise believe their USCI was effective may speak out about the need for patient choice. Advocates and policy makers will need to engage respectfully, and be careful not to appear paternalistic or obstructionist when striving for change.

## Conclusions: the need for bureaucratic will and grassroots efforts

There are many legal and regulatory tools that can be used to protect the public from the misrepresentations and harms associated with USCIs. To date, Health Canada and the Competition Bureau have been slow to act – though the recent Health Canada declaration and public warning is a welcomed move – and professional regulators seem hesitant to restrict the ability of their members to offer these interventions. Overall, Canadian regulators have been more hesitant to respond to this issue than their counterparts in several other nations [20, 21, 23–25, 27, 29]. The reasons for inaction are likely complex, including a combination of uncertainty regarding both the role and jurisdiction of the various regulatory authorities. But given that all of the mentioned regulators clearly have legal authority to respond – as we have seen in other countries – part of the problem must be associated with a simple lack of political will.

To some degree, the above noted regulatory tools are complaint driven. That is, we need individuals and organizations to engage entities like Health Canada, the Competition Bureau and provincial regulators with their concerns about this growing problem. There is a need for grassroots action to stir more robust policy and enforcement responses. While we recognize a lack of resources may be partly to blame for the inadequate regulatory response, creating more awareness – through the media, social media, professional/scientific organizations and political channels – of the scope of the problem, the lack of good science and the potential for harm may help to generate political momentum for change.

Health professionals and laypeople can make a difference by contacting the various institutions we have mentioned to voice their displeasure over insufficient efforts to curb USCIs (see the list of methods below). Even more importantly, individuals who have undergone USCIs and experienced adverse effects, or feel they were misled by related advertising, should file official complaints with professional regulators, the Competition Bureau and Health Canada. It is only by convincing key institutions and leaders of the severity of this problem that we can hope to create positive change.

### How to take action against DTC unproven stem cell therapies

#### Health Canada

- Submit official complaints about USCIs to Health Canada using the online Health Product Complaint FRM-0317 (http://healthycanadians.gc.ca/apps/radar/MD-IM-0005.08.html).
- Contact in writing Health Canada’s Health Product Compliance Directorate (HPC-CPSCorrespondence@hc-sc.gc.ca) to urge Health Canada to increase enforcement.

#### Competition bureau

- Submit official complaints about USCI-related advertising claims to the Competition Bureau using their online Complaint Form (http://www.competitionbureau.gc.ca/eic/site/cb-bc.nsf/frm-eng/GH%C3%89T-7TDNA5).
- Contact the Competition Bureau by phone or mail (http://www.competitionbureau.gc.ca/eic/site/cb-bc.nsf/eng/00157.html) to urge it to take more action.

#### Professional regulation

- Submit general or adverse event-related complaints to provincial self-regulatory colleges, especially those of physicians and surgeons and naturopaths. College websites have information on how to submit complaints, as do the websites of most other self-regulatory bodies for other health professions.

#### Litigation

- Publicize and litigate adverse events, misrepresentation and/or fraud if you have received a direct-to-consumer USCI.

#### Legislative or political action

- Contact your local MP and demand action on USCIs, through further changes to Health Canada policy or other means.
- Contact your local MLA and demand increased oversight for self-regulating bodies, or policy change forcing those bodies to ban members from offering USCIs.

## Data Availability

Not applicable.

## Abbreviations

CMA Code: Canadian Medical Association Code of Ethics and Professionalism
CTO Regulations: Safety of Human Cells, Tissues and Organs for Transplantation Regulations
FD Regulations: Food and Drug Regulations
FDA: Food and Drug Administration (United States)
MD Regulations: Medical Device Regulations
USCI: Unproven Stem Cell Intervention

## References

[1] Turner L, Knoepfler P (2016). Selling stem cells in the USA: assessing the direct-to-consumer industry. Cell Stem Cell.

[2] Turner L (2018). Direct-to-consumer marketing of stem cell interventions by Canadian businesses. Regen Med.

[3] Food and Drug Administration. FDA warns about stem cell therapies; 2017. https://www.fda.gov/forconsumers/consumerupdates/ucm286155.htm. Accessed 18 Dec 2018.

[4] Crowe K. Unapproved stem cell therapies on the market in Canada. CBC News. 2017. https://www.cbcca/news/health/stem-cell-private-clinic-health-canada-osteoarthritis-14401391 Accessed 19 Dec 2018.

[5] Government of Canada. Guidance document: preparation of clinical trial applications for use of cell therapy products in humans; 2015. https://www.canada.ca/en/health-canada/services/drugs-health-products/drug-products/applications-submissions/guidance-documents/clinical-trials/guidance-document-preparation-clinical-trial-applications-use-cell-therapy-products-humans.html. Accessed 19 Dec 2018.

[6] Sipp D, Caulfield T, Kaye J, Barfoot J, Blackburn C, Chan S (2017). Marketing of unproven stem cell–based interventions: a call to action. Sci Transl Med.

[7] McLean AK, Stewart C, Kerridge I (2015). Untested, unproven, and unethical: the promotion and provision of autologous stem cell therapies in Australia. Stem Cell Res Ther.

[8] Rubin R (2018). Unproven but profitable: the boom in US stem cell clinics. JAMA..

[9] Grady D. 12 People Hospitalized With Infections From Stem Cell Shots. New York Times. 2018. https://www.nytimes.com/2018/12/20/health/stem-cell-shots-bacteria-fda.html. Accessed 7 Jan 2019.

[10] Bauer G, Elsallab M, Abou-El-Enein M (2018). Concise review: a comprehensive analysis of reported adverse events in patients receiving unproven stem cell-based interventions. Stem Cells Transl Med.

[11] Daley GQ (2017). Polar extremes in the clinical use of stem cells. N Engl J Med.

[12] Snyder Jeremy, Turner Leigh, Crooks Valorie A. (2018). Crowdfunding for Unproven Stem Cell–Based Interventions. JAMA.

[13] International Society for Stem Cell Research (2016). Guidelines for stem cell research and clinical translation.

[14] Turner L (2018). The US direct-to-consumer marketplace for autologous stem cell interventions. Perspect Biol Med.

[15] Sugarman J, Barker RA, Kerridge I, Lysaght T, Pellegrini G, Sipp D (2018). Tackling ethical challenges of premature delivery of stem cell-based therapies: ISSCR 2018 annual meeting focus session report. Stem cell reports.

[16] Ogbogu U, Rachul C, Caulfield T (2013). Reassessing direct-to-consumer portrayals of unproven stem cell therapies: is it getting better?. Regen Med.

[17] Caulfield T, Sipp D, Murry CE, Daley GQ, Kimmelman J (2016). Confronting stem cell hype. Science..

[18] Knoepfler PS (2018). Too much carrot and not enough stick in new stem cell oversight trends. Cell Stem Cell.

[19] Horner C, Tenenbaum E, Sipp D, Master Z (2018). Can civil lawsuits stem the tide of direct-to-consumer marketing of unproven stem cell interventions. NPJ Regenerative medicine.

[20] U.S. Food and Drug Administration. Statement from FDA Commissioner Scott Gottlieb, M.D. on the FDA’s new policy steps and enforcement efforts to ensure proper oversight of stem cell therapies and regenerative medicine. 2018. https://www.fda.gov/NewsEvents/Newsroom/PressAnnouncements/ucm573443.htm. Accessed 8 Jan 2019.

[21] U.S. Food and Drug Administration. FDA seeks permanent injunctions against two stem cell clinics; 2018. https://www.fda.gov/newsevents/newsroom/pressannouncements/ucm607257.htm. Accessed 8 Jan 2019.

[22] U.S. Food and Drug Administration. FDA warns StemGenex biologic laboratories LLC of illegally marketing an unapproved cellular product manufactured in a facility with significant manufacturing violations, putting patients at risk. 2018. https://www.fda.gov/NewsEvents/Newsroom/PressAnnouncements/ucm625727.htm. Accessed 1 Feb 2019.

[23] Federal Trade Commission (2018). FTC stops deceptive health claims by a stem cell therapy clinic.

[24] Joseph A. Feds crack down on stem cell clinics that touted autism treatments, blindness cures. STAT. 2018. https://www.statnews.com/2018/10/18/feds-crack-down-on-stem-cell-clinics-that-touted-autism-treatments-blindness-cures/. Accessed 7 Jan 2019.

[25] Federal Trade Commission (2018). Stemming unproven stem cell therapy claims.

[26] Munsie M, Rasko J. Private clinics’ peddling of unproven stem cell treatments is unsafe and unethical. The Conversation. 2017. https://theconversation.com/private-clinics-peddling-of-unproven-stem-cell-treatments-is-unsafe-and-unethical-80608. Accessed 7 Jan 2019.

[27] Australian Government. Regulation of autologous cell and tissue products: Department of Health Therapeutic Goods Administration; 2017. https://www.tga.gov.au/media-release/regulation-autologous-cell-and-tissue-products. Accessed 7 Jan 2019.

[28] Australian Govenrment. Autologous human cells and tissues products regulation: Department of Health Therapeutic Goods Administration; 2018. https://www.tga.gov.au/autologous-human-cells-and-tissues-products-regulation. Accessed 1 Feb 2019.

[29] August JW, Payton M, Jones T (2018). State task force forming to investigate stem cell clinics.

[30] *Food and Drugs Act*, RSC 1985, c F-27.

[31] Food and Drug Regulations, CRC, c 870.

[32] Medical Devices Regulations, SOR/98–282.

[33] Safety of Human Cells, Tissues and Organs for Transplantation Regulations, SOR/2007–118.

[34] Viswanathan S, Bubela T (2015). Current practices and reform proposals for the regulation of advanced medicinal products in Canada. Regen Med.

[35] Health Canada Policy Position Paper – Autologous Cell Therapy Products. 2019. https://www.canada.ca/en/health-canada/services/drugs-health-products/biologics-radiopharmaceuticals-genetic-therapies/applications-submissions/guidance-documents/cell-therapy-policy.html. Accessed 30 May 2019.

[36] Von Tigerstrom B (2015). Revising the regulation of stem cell-based therapies: critical assessment of potential models. Food & Drug LJ.

[37] Chisholm J, von Tigerstrom B, Bedford P, Fradette J, Viswanathan S (2017). Workshop to address gaps in regulation of minimally manipulated autologous cell therapies for homologous use in Canada. Cytotherapy..

[38] Code of Federal Regulations, Title 21. 21 Part 1271 Human Cells, Tissues, and Cellular and Tissue-Based Products (rev. in 2014).

[39] Competition Act, RSC 1985, c C-34.

[40] Competition Bureau. False and Misleading Representations and Deceptive Marketing Practices under the Competition Act. 2015. http://www.competitionbureau.gc.ca/eic/site/cb-bc.nsf/eng/03133.html. Accessed 3 Dec 2018.

[41] Canada (Commissioner of Competition) v. Chatr Wireless Inc. 2013 ONSC 5315 at para 295. http://www.canlii.org/en/on/onsc/doc/2013/2013onsc5315/2013onsc5315.html. Accessed 14 Feb 2019.

[42] Stem Cell Network. https://stemcellnetwork.ca/. Accessed 23 Jan 2019.

[43] Murdoch B, Zarzeczny A, Caulfield T (2018). Exploiting science? A systematic analysis of complementary and alternative medicine clinic websites’ marketing of stem cell therapies. BMJ Open.

[44] College of Physicians and Surgeons of Ontario. Policies: Complementary / Alternative Medicine. Updated 2011. https://www.cpso.on.ca/Physicians/Policies-Guidance/Policies/Complementary-Alternative-Medicine. Accessed 17 July 2019.

[45] Collège des Médecins du Québec. Code of Ethics of Physicians. Pursuant to: Medical Act (R.S.Q., c. M-9, s. 3) Professional Code (R.S.Q., c. C-26, s. 87). http://www.cmq.org/publications-pdf/p-6-2015-01-07-en-code-de-deontologie-des-medecins.pdf?t=1525377131660. Accessed 14 Feb 2019.

[46] College of Physicians and Surgeons of British Columbia. Practice Standards and Professional Guidelines. https://www.cpsbc.ca/for-physicians/standards-guidelines. Accessed 14 Feb 2019.

[47] College of Physicians and Surgeons of Alberta. Standard of practice: complementary and alternative medicine; 2014. http://www.cpsa.ca/standardspractice/complementary-alternative-medicine/. Accessed 14 Feb 2019.

[48] Ontario (College of Physicians and Surgeons of Ontario) v. Hanson, 2009 ONCPSD 19 (CanLII).

[49] Ontario (College of Physicians and Surgeons of Ontario) v. Kooner, 2008 ONCPSD 16 (CanLII).

[50] College of Physicians and Surgeons of Alberta. Standard of practice – advertising; 2015. http://www.cpsa.ca/standardspractice/advertising/. Accessed 23 Jan 2019.

[51] College of Physicians & Surgeons of Nova Scotia. Professional Standards Regarding Advertising and Public Communications by Physicians. 2018. https://cpsns.ns.ca/wp-content/uploads/2017/10/Advertising-and-Public-Communications-by-Physicians-Standards.pdf. Accessed 23 Jan 2019.

[52] Canadian Medical Association. CMA code of ethics and professionalism; 2018. http://policybase.cma.ca/dbtw-wpd/Policypdf/PD19-03.pdf. Accessed 23 Jan 2019.

[53] *Medicine Act*, 1991, SO 1991, c 30.

[54] *The Medical Act*, CCSM c M90.

[55] *Health Professions Act,* RSA 2000, c H-7.

[56] *Health Professions Act,* RSBC 1996, c 183.

[57] College of Physicians and Surgeons of Alberta. Stem cell regenerative therapy: Standards & Guidelines; 2018. http://www.cpsa.ca/wp-content/uploads/2018/02/Stem-Cell-Regenerative-Therapy-Standards.pdf. Accessed 12 Dec 2018.

[58] ter Neuzen v. Korn, 1995 127 D.L.R. (4th) 577 (S.C.C.).

[59] Caulfield T (2012). Commentary: the law, unproven CAM and the two-hats fallacy: guest editorial. Focus Altern Complement Ther.

[60] Dickens BM (2003). Malpractice liability implications of pace- maker and defibrillator guidelines in Canada. Card Electrophysiol Rev.

[61] International Society for Stem Cell Research (2018). Letter to the Honourable Ginette Petitpas Taylor, minister of health.

